# Gastrointestinal bleeding is associated with renal prognosis in adult patients with IgA vasculitis with nephritis

**DOI:** 10.1002/jgf2.285

**Published:** 2019-10-15

**Authors:** Yoshinosuke Shimamura, Takuto Maeda, Keitaro Nishizawa, Yayoi Ogawa, Hideki Takizawa

**Affiliations:** ^1^ Department of Nephrology Teine Keijinkai Medical Center Sapporo Japan; ^2^ Hokkaido Renal Pathology Center Sapporo Japan

**Keywords:** clinical remission, end‐stage kidney disease, gastrointestinal bleeding, IgA vasculitis with nephritis

## Abstract

**Background:**

Although the prediction of renal prognosis in patients with IgA vasculitis with nephritis (IgAVN) is important, the association between gastrointestinal bleeding (GIB) and its renal prognosis is unknown. This study investigated the effect of GIB on the progression to end‐stage kidney disease (ESKD) in patients with IgAVN.

**Methods:**

We compared the clinicopathological findings at diagnosis, therapy, and clinical outcomes between 10 patients with GIB and 20 patients without GIB in 30 patients with IgAVN aged ≥18 years at the renal biopsy. The primary outcome was the incidence of ESKD. Secondary outcomes included clinical remission and all‐cause mortality. The outcomes and factors affecting the progression to ESKD were evaluated using the Kaplan‐Meier method with log‐rank test and Cox proportional hazards models.

**Results:**

End‐stage kidney disease, clinical remission, and deaths from any related cause occurred in 6, 17, and 2 patients, respectively. In Kaplan‐Meier analyses, the GIB group showed a higher incidence of ESKD (50% vs 5%, *P* = .003) and a lower incidence of clinical remission (20% vs 75%, *P* = .003). Although the numbers were not statistically significant, this group tended to have a greater number of deaths than the non‐GIB group (7% vs 0%, *P* = .07). In a multivariable Cox model adjusted for hypertension and urinary proteinuria, GIB could not demonstrate a significant association with ESKD (hazard ratio, 4.51; 95% confidence interval, 0.39‐52.7; *P* = .23).

**Conclusion:**

IgAVN with GIB has worse renal outcome, but GIB does not have a statistically significant association with progression to ESKD.

## Graphical abstract

We performed a retrospective study on a patient cohort with IgAVN to compare the outcomes between the GIB and non‐GIB groups. This study indicated that GIB is associated with the incidence of ESKD and clinical remission.

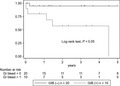

## 1 INTRODUCTION

Immunoglobulin A vasculitis (IgAV), formerly known as Henoch‐Schönlein purpura, is an immune complex vasculitis affecting small vessels with dominant IgA deposits, involving the skin, joints, gastrointestinal tract, and kidney.^1^ Although it primarily occurs in children, 25%‐30% of patients with IgAV are adults^2^; recent studies in Japan showed that the disease has a bimodal age distribution, which peaks in 20‐ to 29‐year‐old and 60‐ to 69‐year‐old individuals.^1^, ^3^, ^4^, ^5^, ^6^, ^7^ IgAV is often involved with the kidneys, which is called IgAV with nephritis (IgAVN): 30%‐50% of children and 45%‐85% of adult cases.^1^, ^3^ They also reported that adult patients with IgAVN have a higher rate of renal involvement and, thus, a greater risk of progression to end‐stage kidney disease (ESKD) than children.^6^, ^7^, ^8^, ^9^ Clinical findings at the time of diagnosis indicated advanced age, baseline renal function, lower serum albumin levels, hypertension, and proteinuria to be risk factors for ESKD.^7^, ^8^, ^10^, ^11^, ^12^ A multicenter retrospective cohort study, using the Japan Renal Biopsy Registry, demonstrated that age older than 65 years and hypoalbuminemia were the independent prognostic factors for a decline in renal function.^13^ Although gastrointestinal bleeding (GIB) because of various causes, such as hemorrhagic erosions and ulcers, is common,^9^, ^14^, ^15^ its impact on renal prognosis in patients with IgAVN remains uncertain. In addition, patients with IgAV often visit primary care physicians with common complaints such as palpable purpura, arthritis, and gastrointestinal symptoms, so that study on the prognosis of IgAV is relevant for general physicians. Therefore, we conducted this retrospective cohort study to investigate the effects of GIB on renal prognosis and mortality; furthermore, we examined the factors that affect the progression to ESKD in adult patients with IgAVN.

## 2 METHODS

### 2.1 Patients

A total of 1085 patients underwent kidney biopsy at the Teine Keijinkai Medical Center, a 650‐bed tertiary reference center, in Sapporo situated at the northern island of Japan between January 1, 2000, and December 31, 2018. Inclusion criteria were as follows: age of 18 years or older; diagnosis of IgAVN between January 1, 2000, and November 30, 2018; treatment with renin‐angiotensin system inhibitors, steroids, or other immunosuppressive regimens for at least a month; follow‐up after at least a month of diagnosis; and consent to enroll in the study. Patients were considered to have IgAVN based on a modification of the classification criteria by the European League Against Rheumatism/the Paediatric Rheumatology European Society (EULAR/PRINTO/PRES): presence of purpura or petechiae with lower limb predominance and unrelated thrombocytopenia, histologically proven small‐vessel vasculitis, histologically proven IgA deposits, and the involvement of at least one organ (from among kidney, joints, or intestinal tract). Patients were excluded from the analysis if they had kidney diseases other than IgAVN between January 1, 2000, and November 30, 2018, and if they refused to enroll in the study. After application of the exclusion criteria, 35 patients were included in our study. This study was conducted according to the Declaration of Helsinki and was approved by the institutional review board of Teine Keijinkai Medical Center (approval no. 2018‐173). The participants in this study provided their written informed consent. The findings of this study follow the checklist for STROBE, that is, Strengthening the Reporting of Observational Studies in Epidemiology (STROBE), for observational studies.^16^ We performed a chart review of patients with IgAVN using the Teine Keijinkai Renal Biopsy Registry (TK‐RBR) at Teine Keijinkai Medical Center and gathered information between January 1, 2000, and December 31, 2018.

### 2.2 Definitions and measurements

The main measurement of exposure in this study was GIB one month before and after the diagnosis of IgAVN, which had been defined as endoscopically proven GIB, melena, and/or hematemesis. Baseline characteristics, as well as laboratory and pathological data, were evaluated by physicians in charge of each patient in every individual case at the time of the initial evaluation. Baseline characteristics of the patients included age, gender, body mass index (body weight [kg]/height^2^ [m^2^]), systolic and diastolic blood pressure, presence/absence of edema, comorbidities (hypertension, diabetes mellitus, liver diseases, endometriosis, uterine fibroids), and prescribed medications (aspirin, warfarin, nonsteroidal anti‐inflammatory drugs). Hypertension was defined as a state in which systolic blood pressure was greater than 140 mm Hg and/or diastolic blood pressure was greater than 90 mm Hg, and antihypertensive drugs were being used as treatment before diagnosis of IgAVN. Diabetes mellitus was defined as the condition in which HbA1c was greater than 6.5% (National Glycohemoglobin Standardization Program) and/or treatment with hypoglycemic drugs was ongoing before diagnosis. Liver disease was defined as the presence of viral hepatitis (hepatitis B virus or hepatitis C virus) or alcoholic liver disease before diagnosis. Information on the presence or absence of endometriosis and uterine fibroids was collected from hospital medical records or databases. Laboratory data collected for the study included values of urine in urine sediment red blood cell counts (≥30 per high‐power field), urinary protein levels, serum red blood cell counts, platelet counts, blood urea nitrogen (BUN), serum creatinine (sCr), estimated glomerular filtration rates (eGFR), serum albumin, serum immunoglobulin A (IgA) levels, complement 3 (C3) levels, complement 4 (C4) levels, and CH50 levels. Estimated glomerular filtration rates were calculated using a modified form of the IDMS‐MDRD Study equation for Japanese individuals. Renal biopsy was performed at the time of diagnosis. Renal specimens were examined by a nephrological pathologist (YO) and were classified based on the International Study of Kidney Disease in Children (ISKDC) classification system. The modalities of initial treatment were recorded, such as the initial oral dose of corticosteroid, steroid pulse therapy (methylprednisolone 0.5 g/d for three consecutive days), along with the number of courses, renin‐angiotensin system inhibitors (RASi), immunosuppressive agents and their descriptions, and tonsillectomy. Here, the RASi included the angiotensin‐converting enzyme inhibitors and angiotensin II receptor antagonists.

The primary outcome of interest that was measured here was the incidence of ESKD, which was indicated by an eGFR less than 15 mL/min/1.73 m^2^ or the initiation of renal replacement therapy, including hemodialysis, peritoneal dialysis, or renal transplant within 5 years of diagnosis. The incidence of ESKD was ascertained from each patient's medical records prepared by the physicians in charge of them. Secondary outcomes recorded here were time to death from any cause and time to clinical remission, which was defined as the disappearance of hematuria and proteinuria. The disappearance of hematuria was defined as a condition with fewer than 5/high‐power field of erythrocytes in sediment or (−) to (±) in dipstick tests. The disappearance of proteinuria was defined by the presence of less than a total of 0.3 g/d of protein in urine samples collected over a 24‐hour period or urine protein/urine creatinine ratio of less than 0.3, when recorded from spot urine or (−) to (±) dipstick tests. The follow‐up period was defined as the time from the initial diagnosis to (a) incidence of ESKD or (b) the last follow‐up visit, whichever came first. Cases in which death occurred before ESKD incidence were not considered for further analysis.

### 2.3 Statistical analyses

We used Student's *t* test or the Wilcoxon rank‐sum test to compare continuous variables based on their distributions, and Fisher's exact test to compare the proportions of categorical variables between the GIB and non‐GIB groups. The normality of the variance for each continuous variable was analyzed using the Shapiro‐Wilk W test. The ISKDC classification was compared between the groups using single‐factor analysis of variance (ANOVA).

We used the Kaplan‐Meier method to estimate incidences of ESKD, clinical remission, and death; the records thus obtained were compared by using the log‐rank tests. Data were censored on December 31, 2018. Patients who were lost till the time of follow‐up were removed at the date of the last contact. Patients who were alive on December 31, 2018, were considered for analysis. We used Cox proportional hazards models to assess the association of GIB and several covariates with the incidence of ESKD; the results were expressed as hazard ratios (HR) with 95% confidence intervals (CI). All the independent variables included in univariable analyses were either categorical (coded as 0/1) or quantitative. Categorical variables included hypertension, RASi, endocapillary proliferation lesions (≥25%), steroid pulse therapy, and GIB. Quantitative variables included age, gender, urinary protein levels, eGFR, and serum red blood cell counts. Multivariable Cox analyses were applied to determine the relationship between the incidence of ESKD and GIB, as well as age, gender, hypertension, and urinary protein levels. Variables included in the multivariable Cox analysis were selected based on previous studies and the results of the univariable analysis. The sample size calculation was based upon the primary outcome, which was estimated to be 50%^17^ in the GIB group and 20%^8^, ^10^, ^11^, ^18^ in the non‐GIB group. With a significance threshold of *P* = .05 in statistical analyses and 80% statistical power, the total sample size was calculated to be 80 after adjusting for 5% attrition, leaving 40 individuals in each group.

We have presented data on continuous variables as means with standard deviations (SD) or medians with associated interquartile ranges, while categorical variables are presented as numbers and percentages. We used the STATA software (version 15.1; StataCorp LLC) to perform statistical analyses. All the reported *P*‐values are two‐sided. We considered a *P*‐value less than .05 to indicate statistical significance.

## 3 RESULTS

### 3.1 Patients

Among the 1085 eligible patients who were registered in the TK‐RBR from January 1, 2000, to November 30, 2018, at the Teine Keijinkai Medical Center, 35 (3.4%) only the patients with IgAVN were included in this study. We excluded three patients who had not completed the one‐month follow‐up requirement and two patients who had missing data. The remaining 30 patients were included in further analyses.

### 3.2 Clinicopathological characteristics and treatment modalities

Baseline clinicopathological characteristics are summarized in Table 1. Ten out of the 30 (33%) patients developed GIB. The median age of patients was 58 years, and the mean body mass index was 24 ± 4.3 kg/m^2^. These statistics were comparable between the two groups (*P* = .059 and .81, respectively). As compared to the patients without GIB, patients with GIB had a higher prevalence of hypertension (70% vs 25%, *P* = .045), higher urinary protein levels (median [IQR], 4.8 [2.3‐7.3] vs 1.3 [0.8‐2.9] g/d, *P* = .011), lower serum red blood cell counts (mean ± SD, 359 ± 93 vs 423 ± 65 ×10^3^/μL, *P* = .003), and lower serum albumin levels (mean ± SD, 2.8 ± 0.7 vs 3.4 ± 0.8 g/dL, *P* = .023). Baseline serum creatinine levels (median [IQR], 1.17 [0.69‐2.49] vs 0.87 [0.61‐1.24] mg/dL, *P* = .38), hemoglobin levels (mean ± SD, 10.8 ± 2.8 vs 11.7 ± 2.3 g/dL, *P* = .58), and serum IgA levels (median [IQR], 296 [277‐460] vs 311 [277‐388] mg/dL, *P* = .72) were comparable between the two groups. Renal biopsy was performed in all patients. According to the ISKDC classification, 7/23 (23%) patients belonged to class II, 16/30 (53%) belonged to class IIIa, and 7/30 (23%) belonged to class IIIb. No patients were assigned to class I, IV, V, or VI.

**Table 1:** Comparison of clinicopathological findings between patients with IgAVN (n = 30) from the GIB and non‐GIB groups

	All (n = 30)	GIB (+) (n = 10)	GIB (−) (n = 20)	*P*‐value
Median age, y (median [25, 75%])	58 [40, 65]	64 [53, 76]	50 [33, 62]	.059
Women, no. (%)	12 (40)	5 (50)	7 (35)	.46
Diabetes, no. (%)	7 (23)	4 (40)	3 (15)	.18
Hypertension, no. (%)	12 (40)	7 (70)	5 (25)	.045
Liver disease, no. (%)	8 (27)	2 (20)	6 (30)	.68
Antiplatelets, no. (%)	6 (20)	2 (30)	2 (15)	.37
Anticoagulants, no. (%)	0	0	0	‐
NSAIDs, no. (%)	0	0	0	‐
Syncope, no. (%)	0	0	0	‐
Heart failure, no. (%)	0	0	0	‐
Systolic BP (mm Hg; mean ± SD)	134 ± 24	141 ± 28	130 ± 22	.27
Diastolic BP (mm Hg; mean ± SD)	79 ± 13	80 ± 15	78 ± 13	.72
Edema, no. (%)	15 (50)	7 (70)	8 (40)	.25
BMI (kg/m^2^; mean ± SD)	24 ± 4.3	24 ± 3.8	24 ± 4.6	.81
Endometriosis, no. (%)	1/12 (8)	1/5 (20)	0/7 (0)	.36
Uterine fibroid, no. (%)	2/12 (17)	1/5 (20)	1/7 (14)	1.0
Blood urea nitrogen (mg/dL; median [25, 75%])	16.4 [12.4, 30]	22.6 [14, 36]	14.5 [12.0, 19.7]	.091
Serum creatinine (mg/dL; median [25, 75%])	0.89 [0.63, 1.5]	1.17 [0.69, 2.49]	0.87 [0.61, 1.24]	.38
Urinary protein (g/d; median [25, 75%])	1.9 [0.82, 4.7]	4.8 [2.3, 7.3]	1.3 [0.8, 2.9]	.011
Sediment RBC ≥ 30/HPF, no. (%)	24 (80)	10 (100)	14 (70)	.074
RBC (10^3^/μL; mean ± SD)	402 ± 80	359 ± 93	423 ± 65	.003
Hemoglobin (10^6^/μL; mean ± SD)	11.1 ± 2.6	10.8 ± 2.8	11.7 ± 2.3	.58
Platelet (10^6^/μL; median [25, 75%])	23.7 [20.7, 31.9]	27 [20, 33.3]	23.7 [21.6, 28.7]	.69
PT‐INR (median [25, 75%])	1.01 [0.96, 1.08]	1.03 [0.98, 1.09]	1.0 [0.95, 1.02]	.17
eGFR (mL/min/1.73 m^2^; median [25, 75%])	64 [26, 91]	23.5 [14, 72]	76.5 [54.5, 92]	.04
Albumin (g/dL; mean ± SD)	3.2 ± 0.8	2.8 ± 0.7	3.4 ± 0.8	.023
IgA (mg/dL; median [25, 75%])	305 [277, 396]	296 [277, 460]	311 [277, 388]	.72
C3 (mg/dL; median [25, 75%])	122 [105, 134]	121 [102, 151]	122 [106, 132]	.98
C4 (mg/dL; median [25, 75%])	29 [26, 40]	37 [28, 42]	28 [25, 36]	.17
CH50 (mg/dL; median [25, 75%])	45 [39, 50]	47 [38, 53]	44 [40, 50]	.86
Steroids, no. (%)	26 (87)	9 (90)	17 (85)	1.0
Initial dose (mg/dL; mean ± SD)	32.6 ± 7.5	35.0 ± 7.9	31.3 ± 7.2	.34
Pulse therapy, no. (%)	15 (50)	5 (50)	10 (50)	1.0
Courses of pulse therapy (n = 1/2/3)	6/0/9	3/0/2	3/0/7	‐‐
RAS inhibitors, no. (%)	11 (30)	5 (50)	6 (30)	.43
Immunosuppressive agents, no. (%)	2 (7)	1 (10)	1 (5)	1.0
ISKDC classification, no. (%)				
I	0	0	0	.47
II	7 (23)	2 (20)	5 (25)	
IIIa	16 (53)	4 (40)	12 (60)	
IIIb	7 (23)	4 (40)	3 (15)	
IV	0	0	0	
V	0	0	0	
VI	0	0	0	

Note

BMI, body mass index; BP, blood pressure; eGFR, estimated glomerular filtration rate; GIB, gastrointestinal bleeding; HPF, high‐power field; ISKDC classification, the International Study of Kidney Disease in Children classification; NSAIDs, nonsteroidal anti‐inflammatory drugs; PT‐INR, prothrombin time internationalized ratio; RBC, red blood cell; SD, standard deviation; RAS inhibitors, renin‐angiotensin system inhibitors.

Table 1 also summarizes treatments between the GIB and non‐GIB groups. A total of 26 out of the 30 (87%) patients received oral corticosteroids with mean initial doses of 35.0 ± 7.9 mg/d in the GIB group and 31.0 ± 7.2 mg/d in the non‐GIB group (*P* = .34); 15/30 (50%) patients received steroid pulse therapy, where 5/10 (50%) patients were from the GIB group and 10/20 (50%) were from the non‐GIB group (*P* = 1.0). The numbers of steroid pulse courses varied between 1 and 3 in each group. The rate of treatment with a selected RASi (5/10 (50%) vs. 6/20 (30%)) did not differ between the groups (*P* = .43). A total of 2 out of 27 (7%) patients received intravenous cyclophosphamide as a part of the initial therapy.

### 3.3 Between‐group comparison of primary and secondary outcomes

During a median follow‐up of 2.6 years, ESKD, clinical remission, and deaths from any cause occurred in 6/30 (20%), 17/30 (57%), and 2/30 (6.7%) of the patients, respectively. Of note was one patient in the non‐GIB group, who developed ESKD. The patient was a 58‐year‐old Japanese man with a history of diabetes, hypertension, hepatitis B virus infection, and ischemic heart disease, who was referred to our institution with a 6‐month history of purpuric lesions in his lower extremities. Laboratory data showed 40.9 mg/dL BUN, 4.73 mg/dL sCr, and eGFR of 16 mL/min/1.73 m^2^ at the time of histological diagnosis of IgAVN.

**Figure 1:**
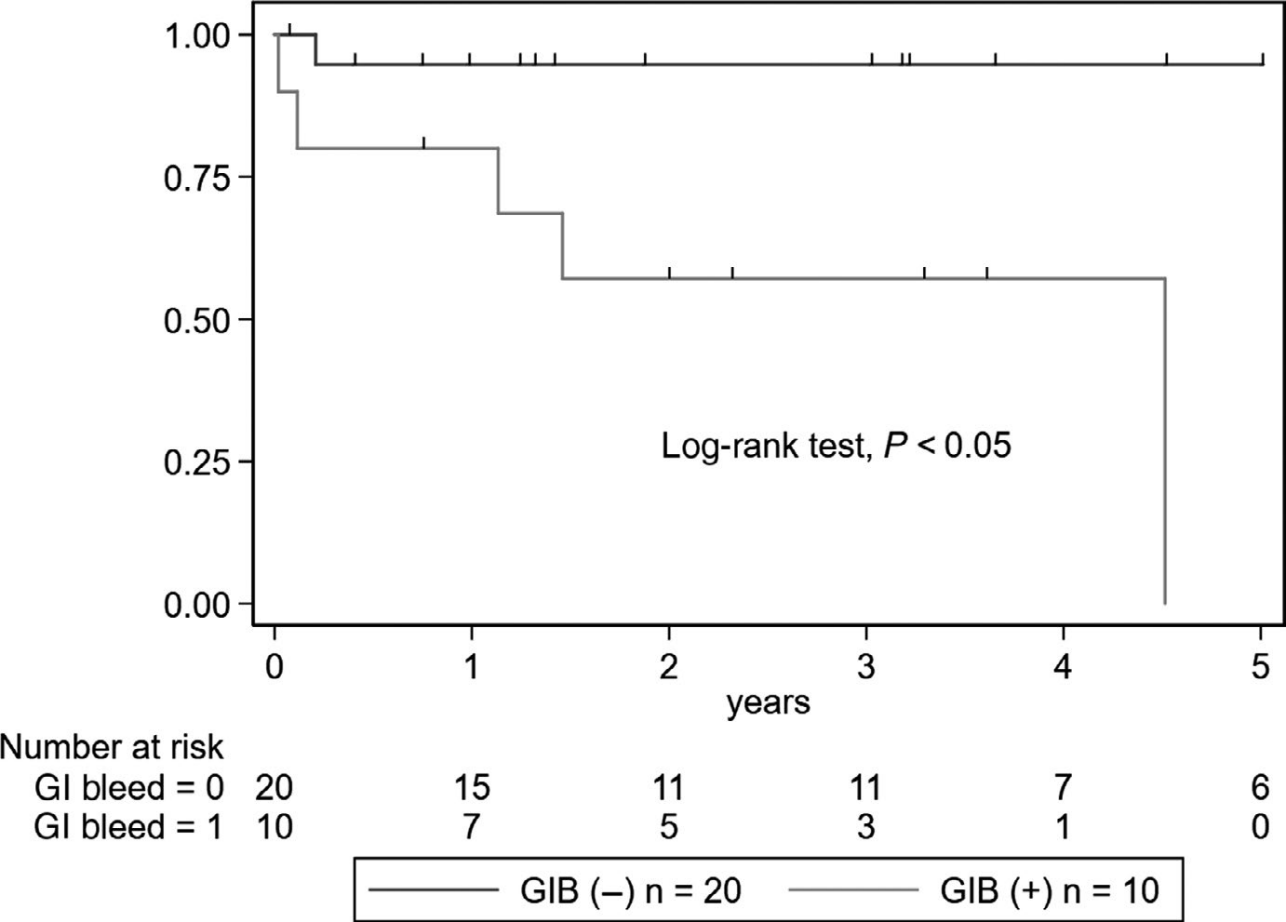
Kaplan‐Meier curves of the progression to ESKD in the GIB and non‐GIB groups

**Figure 2:**
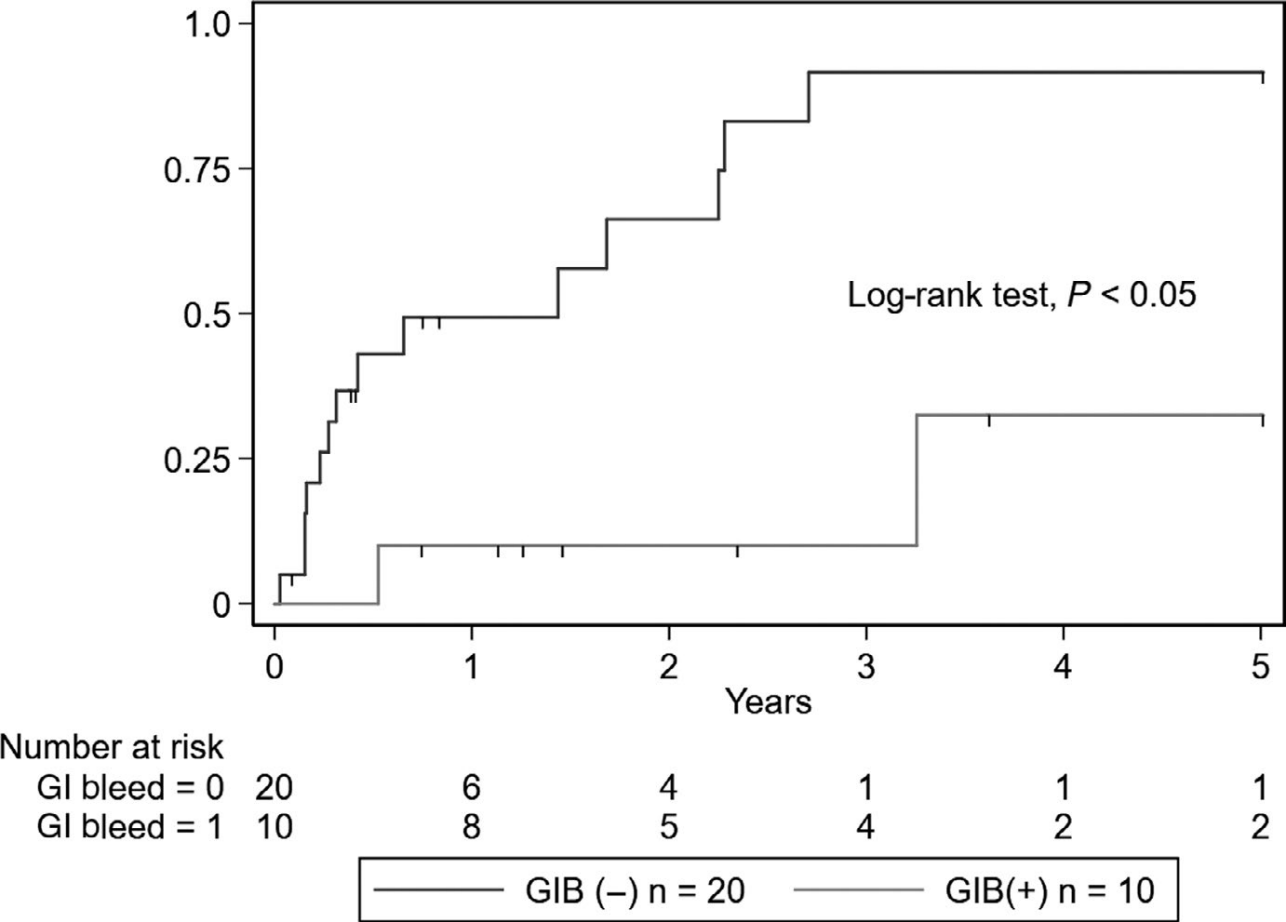
Kaplan‐Meier curves of clinical remission in the GIB and non‐GIB groups

The incidence of ESKD was significantly higher in the GIB group (five events, 50%) than in the non‐GIB group (one event, 5%) (log‐rank test, *P* = .003) (Figure 1). Additionally, the incidence of clinical remission was significantly lower in the GIB group (2 events, 20%) than in the non‐GIB group (15 events, 75%) (log‐rank test, *P* = .003) (Figure 2). A total of 2 (6.7%) deaths occurred, both in the GIB group as a result of hemorrhagic shock secondary to massive GIB. However, the mortality rate was not significantly different between the two groups (log‐rank test, *P* = .07).

### 3.4 Factors affecting progression to ESKD

As per the univariable analysis with Cox proportional hazards models (Table 2), GIB (HR, 13.3; 95% CI, 1.50‐117), urinary protein levels (HR, 1.39; 95% CI, 1.12‐1.73), and hypertension (HR, 9.58; 95% CI, 1.11‐82.6) were factors that were associated with a significantly increased risk of progression to ESKD. Table 3 describes multivariable analyses with Cox proportional hazards models. In Model 1, which was adjusted for age and gender, GIB remained independently associated with progression to ESKD (HR, 22; 95% CI, 1.63‐296). However, GIB lost this association after further adjustment for urinary protein levels and hypertension (Models 2 and 3).

**Table 2:** Univariable analysis of factors affecting the progression to ESKD (n = 30)

	Hazard ratio	95% CI	*P*‐value
GIB	13.3	1.50‐117	.02
Urine protein	1.39	1.12‐1.73	.003
Hypertension	9.58	1.11‐82.6	.04
Age	1.06	0.99‐1.12	.08
Gender	1.57	0.29‐8.60	.606
Red blood cells	0.99	0.98‐1.0	.165
Steroid pulse therapy	0.96	0.16‐5.79	.965
RAS inhibitors	4.46	0.81‐24.6	.086
eGFR	0.81	0.64‐1.03	.082

Note

ESKD, end‐stage kidney disease; GIB, gastrointestinal bleeding; RAS inhibitors, renin‐angiotensin system inhibitors; eGFR, estimated glomerular filtration rate.

**Table 3:** Multivariable analysis of factors affecting the progression to ESKD (n = 30)

	Hazard ratio	95% CI	*P*‐value
Model 1			
GIB	22	1.63‐296	0.02
Age	1.1	0.98‐1.23	0.102
Gender	14.4	0.66‐312	0.09
Model 2			
GIB	9.56	0.75‐123	0.083
Urine protein	1.27	0.91‐1.78	0.162
Model 3			
GIB	4.51	0.39‐52.7	0.23
Hypertension	14.5	0.43‐494	0.137

Note

Model 1, adjusted for age and gender; Model 2, adjusted for Model 1, urine protein; Model 3, adjusted for Model 2 and hypertension.

## 4 DISCUSSION

This study investigated the clinical impacts of GIB in adult patients with IgAVN and identified the factors that affect progression to ESKD. The results showed that patients who developed GIB were at a higher risk of ESKD with lower chances of clinical remission. Although the relationship was not statistically significant, the patients with GIB tended to have more deaths than those without GIB. Although GIB is not independently associated with progression to ESKD with adjustment for covariates, our results suggest that assessment of GIB may be an effective method to identify patients with IgAVN who are at a high risk of ESKD.

The renal prognosis in adult patients with IgAVN was worse than that in children.^9^ Previous studies have shown that it ranged from 9% to 27% because of substantial heterogeneity across studies, as well as limitations in study designs, patient populations, and definition of outcomes.^8^, ^10^, ^11^, ^18^ A more recent study, using the Japan Renal Biopsy Registry, reported that the rate of decline in renal function (defined as a 50% increase in sCr from baseline or ESKD with renal replacement therapy) was 21.7% in Japanese patients with IgAVN.^13^ In the present study, 20% of the patients reached ESKD in the 5‐year follow‐up period, comparable to results from the previous study.^18^ Additionally, our results from the univariable analysis are in line with previous studies, demonstrating that urinary protein levels and hypertension are independent predictors of renal prognosis in patients with IgAVN.^10^, ^12^, ^18^

We found out that patients with GIB are more likely to develop ESKD and less likely to reach clinical remission than those without GIB. This may be biologically plausible because GIB can cause anemia and subsequent hypo‐oxygenation of the kidney, contributing to the development of ESKD.^19^ Although GIB is one of the most acute life‐threatening clinical manifestations,^9^ only a few studies have reported an association between GIB and long‐term progression to ESKD. A recent retrospective cohort study in France^6^ reported that 43/137 (31%) patients with IgAV had GIB; however, they did not investigate the effect of GIB on the renal prognosis as we did in our study.

This study showed that the GIB group tended to have higher mortality than the non‐GIB group, although the difference was not statistically significant between the groups. This may simply be because of the small sample size (type II error). Interestingly, we observed a relatively higher mortality of 2/30 (6.7%) patients than that reported in prior studies^6^, ^18^ despite aggressive treatment with high‐dose corticosteroids. The reasons remain unclear, but one possible explanation may be related to the inclusion of more severe cases in our study: The baseline sCr levels (1.21 ± 0.92 mg/dL vs 0.91 ± 0.46 mg/dL) and the percentage of urine protein, that is, >3 g/d (32% vs 23%), were higher in our cohort than in the study performed using the Japan Renal Biopsy Registry.^13^

We observed that GIB has a statistically significant association with ESKD with an adjustment for patients’ age and gender; however, this result should be interpreted cautiously because it has a wide 95% confidence interval. Besides, GIB was not statistically significant after further adjustment with urinary protein levels and hypertension. Therefore, we could not conclude that GIB is an independent prognostic factor of ESKD in this study. Baseline characteristics, including age, BUN, and sCr, were not included in the multivariable analysis because they were similar in the two groups. In addition, we did not include ISKDC classification and crescentic lesions in the Cox proportional hazards models because the association between ISKDC classification and renal prognosis has not been observed previously in adult patients with IgAVN^12^, ^20^; furthermore, a recent retrospective cohort study reported that the proportion of crescents is not associated with a higher risk of ESKD.^21^

Most previous studies were performed on pediatric patients and have reported mixed results for the effectiveness of corticosteroids and other immunosuppressive agents, partly because of the differences in the sample populations and definitions of clinical outcomes. While several studies have reported the efficacy of prednisolone therapy in treating renal symptoms^8^, ^22^, ^23^, a recent prospective study reported that there is no difference in the renal outcomes between the prednisolone‐treated and nontreated patients.^24^ Therefore, we did not conduct a comparison of therapeutic effects of different treatments in this study because of its retrospective nature. To evaluate the effectiveness of corticosteroids and other immunosuppressive agents, large‐scale prospective studies are required.

The present study had several limitations. First, background characteristics were different between patients with positive and negative GIB. We tried to control for confounding factors using multivariable analysis to evaluate the prognostic factors; however, unmeasured confounders may still exist. Second, the retrospective study design was limited to the interpretation of our results. Third, the sample size of the present study might be insufficient to evaluate the influence of GIB on renal prognosis. The sample size also restricted the construction of models incorporating all potential confounding factors, especially BUN and sCr, and failed to introduce corrections in independent variables for multiple comparisons. Fourth, the log‐rank tests in our analyses were not adjusted for potential confounders; therefore, our results should be interpreted with caution. Fifth, the study population consisted solely of Japanese patients, so these findings might not be generalizable to other ethnic groups. Validation studies in other settings should be performed. Despite these limitations, this was the first report to assess the association of gastrointestinal bleeding and renal prognosis in patients with IgAVN. Finally, we provide univariable analyses of ESKD, but we were unable to perform robust multivariable analyses for the outcome because of the limited number of events.^25^ Prospective enrollment and multicenter data collection from the time of diagnosis would have been ideal but are difficult to achieve with a single‐center study.

In conclusion, we performed a retrospective study on a patient cohort with IgAVN to compare the outcomes between the GIB and non‐GIB groups. This study indicated that GIB is associated with the incidence of ESKD, as well as clinical remission in patients with IgAVN, but it failed to be identified as an independent predictor of ESKD. Although these findings should be interpreted in the context of the small sample size, GIB may be a useful indicator for identification of patients with IgAVN at a high risk of ESKD.

## CONFLICT OF INTERESTS

The authors have stated explicitly that there are no conflicts of interest in connection with this article.

## AUTHOR CONTRIBUTIONS

YS and HT conceived and designed the study and drafted the manuscript; YS, TM, KN, HT, and YO acquired the data; YS carried out analysis and interpretation of data and statistical analysis; and HT and OY critically revised the manuscript. Human and animal rights: All procedures involving human participants were designed in accordance with the ethical standards of the institutional and/or national research committee at which the studies were conducted (IRB approval number: 2018‐173) and followed the 1964 Helsinki Declaration and its later amendments or comparable ethical standards. Informed consent: Informed consent was obtained from all participants included in the study individually.
